# Dual-Light-Responsive Fe-Doped Covalent Organic Framework-Functionalized SiO_2_ Nanofibrous Membrane for Synergistic Photothermal and Photodynamic Inactivation of Multidrug-Resistant Bacteria

**DOI:** 10.3390/pharmaceutics18060715

**Published:** 2026-06-10

**Authors:** Ting Zou, Lanlan Ni, Keqiang Xu, Yi Chang

**Affiliations:** 1 Key Laboratory for Advanced Technology in Environmental Protection of Jiangsu Province, Yancheng Institute of Technology, Yancheng 224051, China; 234003011030@stu.ycit.edu.cn (T.Z.); 244003011038@stu.ycit.edu.cn (L.N.); 2 Department of Clinical Laboratory, Affiliated Hospital 6 of Nantong University, Yancheng Third People’s Hospital, Yancheng 224001, China

**Keywords:** multidrug-resistant bacteria, covalent organic framework, electrospun nanofibrous membrane, photodynamic therapy, photothermal therapy

## Abstract

**Background/Objectives**: The rapid emergence of multidrug-resistant (MDR) bacteria has increased the demand for non-antibiotic antibacterial strategies. Although photothermal therapy (PTT) and photodynamic therapy (PDT) are promising alternatives, each modality alone may show limited antibacterial efficacy. This study aimed to construct a flexible dual-light-responsive nanofibrous membrane integrating PTT and PDT for improved in vitro antibacterial activity against MDR bacteria. **Methods**: A silica nanofibrous membrane (SNF) was prepared by electrospinning followed by calcination. An Fe-doped sulfonated TpPa covalent organic framework (SCOF-Fe) was then grown in situ on the SNF surface via an interfacial diffusion strategy to obtain SNF@SCOF-Fe. The membrane was characterized in terms of morphology, structure, optical absorption, photothermal performance, Fe loading, Fe leaching, and reactive oxygen species (ROS) generation. In vitro antibacterial activity against supplier-reported MDR *Escherichia coli* (*E. coli*) and methicillin-resistant *Staphylococcus aureus* (*MRSA*) was evaluated under 420 nm, 808 nm, and dual-light (420 + 808 nm) irradiation. **Results**: Fe doping broadened the optical absorption of the COF-functionalized membrane into the near-infrared region and improved its photothermal response. Under dual-light irradiation, SNF@SCOF-Fe generated singlet oxygen, superoxide radicals, and hydroxyl radicals, together with a greater temperature increase than the undoped membrane. Within 15 min, SNF@SCOF-Fe achieved antibacterial rates of 99.29% against *E. coli* and 99.62% against *MRSA*. In addition, controlled dual-light cytocompatibility testing yielded 78.76% viability in L929 fibroblasts and 82.86% viability in MC38 murine colon carcinoma cells after SNF@SCOF-Fe treatment. **Conclusions**: SNF@SCOF-Fe combines dual-light-triggered photothermal heating and ROS generation within a flexible nanofibrous membrane and demonstrated effective in vitro antibacterial activity against two representative resistant bacteria. These findings support further investigation of SNF@SCOF-Fe as a light-responsive antibacterial membrane in relevant in vitro and in vivo models.

## 1. Introduction

The discovery and clinical application of antibiotics revolutionized the treatment of bacterial infections [1]. However, their widespread and often inappropriate use has accelerated the emergence and dissemination of multidrug-resistant (MDR) bacteria, which now pose a major global public health threat [2]. Against this background, the development of alternative antibacterial strategies that do not rely on conventional antibiotics and are less likely to induce resistance has become increasingly important [3].

Among emerging non-antibiotic approaches, light-triggered antibacterial therapies, especially photothermal therapy (PTT) and photodynamic therapy (PDT), have attracted considerable attention because of their broad-spectrum antibacterial potential and low propensity to induce resistance [4,5]. PTT relies on photothermal agents that convert absorbed light energy into localized heat, thereby disrupting bacterial membranes, denaturing proteins, and damaging intracellular biomolecules [6]. Despite its effectiveness, PTT alone often requires relatively high temperatures to achieve satisfactory sterilization, and prolonged or excessive heating may cause inflammation and thermal injury to surrounding healthy tissues [7]. In contrast, PDT uses photosensitizers to generate reactive oxygen species (ROS), such as singlet oxygen (^1^O_2_), superoxide radicals (·O_2_^−^), and hydroxyl radicals (·OH), under light irradiation [8]. These ROS induce oxidative damage to bacterial membranes and intracellular components. Nevertheless, the efficacy of PDT is often limited by light penetration, local oxygen availability, and the short diffusion distance of ROS [9]. Moreover, excessive ROS generation, although beneficial for bacterial killing, may also cause collateral damage to normal tissues [10]. Therefore, achieving effective antibacterial activity while minimizing thermal and oxidative side effects remains a central challenge.

Recent studies have shown that combining PTT and PDT can significantly improve antibacterial performance at relatively mild temperatures [11,12]. Mild hyperthermia can increase membrane permeability and facilitate ROS penetration, whereas oxidative damage can weaken bacterial tolerance to thermal stress [13,14]. As a result, the integration of PTT and PDT offers an effective route toward synergistic antibacterial therapy [15,16,17]. The development of such synergistic platforms depends on multifunctional materials with strong light-harvesting ability, stable ROS generation, an evident photothermal response, and favorable biocompatibility. Therefore, a key materials-design question is how to construct an integrated platform that can simultaneously support visible-light-triggered ROS generation and near-infrared-triggered photothermal conversion while maintaining a membrane format suitable for interfacial antibacterial evaluation.

Covalent organic frameworks (COFs), a class of crystalline porous polymers constructed from light elements through robust covalent bonds, have emerged as promising candidates because of their high structural tunability, large specific surface area, thermal stability, and chemical robustness [18,19,20]. In particular, two-dimensional COFs with extended π-conjugated backbones exhibit strong visible-light absorption, making them attractive for photoresponsive biomedical materials [21]. TpPa-based COFs are especially interesting because their conjugated framework and visible-light activity enable ROS generation under irradiation [22]. However, the intrinsic band structure of TpPa-based systems still limits their absorption in the near-infrared (NIR) region, resulting in an insufficient NIR-triggered photothermal response and thereby constraining their use in combined PTT/PDT systems. Thus, pristine TpPa-based COFs are generally more favorable for visible-light-driven PDT than for efficient NIR-mediated PTT, which limits their ability to achieve wavelength-separated and cooperative PDT/PTT antibacterial action.

Given the highly tunable electronic structure of COFs, rational metal coordination engineering offers a viable strategy to address this limitation [23]. Incorporation of transition-metal sites, particularly Fe species, has been reported to regulate the band structure of COFs, broaden their spectral response, and facilitate charge separation [24,25]. For example, Fe^3+^ doping in covalent triazine-based frameworks was shown to narrow the band gap, red-shift the absorption edge, improve conductivity, and promote charge separation, with Fe^3+^ acting as an “electron relay” during photocatalytic processes [25]. In addition, porphyrin-based Fe-COFs have been reported to exhibit strong visible/NIR absorption, photothermal conversion, singlet oxygen generation, and peroxidase-like activity for ROS production, supporting the feasibility of Fe-containing COFs as phototherapeutic antibacterial platforms [24]. In sulfonated TpPa COFs, abundant hydroxyl, imine, and sulfonic acid-related moieties can serve as coordination sites for metal ions, enabling stable anchoring of Fe species. The introduction of Fe is therefore expected to create additional metal-to-ligand charge-transfer pathways, extend light absorption toward the NIR region, and improve charge-carrier dynamics. In addition, Fe sites may promote the formation of highly reactive ·OH species and further strengthen the overall antibacterial effect. These considerations provide the rationale for using Fe coordination to modulate the optical/electronic properties of TpPa-based COFs and to enhance their dual-light-responsive PDT/PTT antibacterial performance.

Although metal-modified COFs have been explored in photocatalysis and antibacterial systems, studies integrating metal-doped COFs into flexible antibacterial membranes with combined PDT and PTT functions remain limited. On the other hand, electrospun nanofibrous membranes are considered attractive substrates for interfacial antibacterial materials because of their high porosity, large specific surface area, good flexibility, and extracellular-matrix-like architecture [26,27]. Among them, SiO_2_ nanofibrous membranes are particularly attractive because of their biocompatibility, mechanical robustness, chemical stability, and readily modifiable surface chemistry [28]. Immobilizing photoactive antibacterial components on such flexible nanofibers may improve the usability of COF-based materials and provide a basis for multifunctional antibacterial membrane design. Therefore, the novelty of this work does not lie in the isolated use of SiO_2_ nanofibers, TpPa-based COFs, or Fe species, but rather in their rational integration into a flexible Fe-doped COF-functionalized nanofibrous membrane capable of dual-light-responsive photothermal/photodynamic antibacterial action.

Herein, we report the fabrication of a flexible Fe-doped COF-functionalized silica nanofibrous membrane, denoted as SNF@SCOF-Fe, by interfacial growth of a sulfonated TpPa COF on an electrospun silica nanofibrous membrane followed by in situ Fe incorporation. The central research question is whether Fe doping can modulate the optical/electronic properties of TpPa-based COFs to enable dual-light-responsive synergistic PDT/PTT antibacterial activity when integrated into a nanofibrous membrane platform. Under dual-light irradiation at 420 and 808 nm, SNF@SCOF-Fe showed an enhanced heating response and sustained generation of multiple ROS, enabling effective synergistic antibacterial activity against two representative resistant bacterial strains. The composite membrane achieved antibacterial rates of 99.29% against *Escherichia coli* (*E. coli*) and 99.62% against methicillin-resistant *Staphylococcus aureus* (*MRSA*) within 15 min while maintaining acceptable cytocompatibility. This study provides a materials-design strategy for dual-light-responsive antibacterial nanofibrous membranes, while further studies involving biofilm-associated bacterial models, tissue compatibility evaluation, and in vivo validation are still required to assess its potential application relevance.

## 2. Materials and Methods

### 2.1. Preparation of the SiO_2_ Nanofibrous Membrane (SNF)

A silica nanofibrous membrane was fabricated by electrospinning followed by calcination. First, two precursor solutions were prepared. For solution A, 3.2 g of poly(vinyl butyral) (PVB, Shanghai Titan Scientific Co., Ltd., Shanghai, China) was dissolved in 12.8 g of absolute ethanol (Sinopharm Chemical Reagent Co., Ltd., Shanghai, China) under magnetic stirring at room temperature until a clear homogeneous solution was obtained. For solution B, 5.0 g of tetraethyl orthosilicate (TEOS, Shanghai Titan Scientific Co., Ltd., Shanghai, China) and 0.05 g of oxalic acid (Sinopharm Chemical Reagent Co., Ltd., Shanghai, China) were added to a mixed solvent containing 1.5 g of deionized water (laboratory-made) and 1.0 g of absolute ethanol, followed by stirring at room temperature for approximately 8 h to ensure sufficient hydrolysis of TEOS. Subsequently, 4.0 g of solution A was slowly added to solution B and further stirred until a transparent and stable electrospinning precursor solution was formed. The precursor solution was then transferred into an electrospinning setup and processed at an applied voltage of 17 kV with a feeding rate of 1.5 mL·h^−1^. The collected precursor membrane was calcined in a muffle furnace (KSL-1200X, Hefei Kejing Materials Technology Co., Ltd., Hefei, China) at 800 °C for 3 h with a heating rate of 2 °C·min^−1^ to remove the organic components and obtain the silica nanofibrous membrane, denoted as SNF.

### 2.2. Preparation of SiO_2_@TpPa-SO_3_H (SNF@SCOF)

An interfacial diffusion method was used to grow TpPa-SO_3_H in situ on the surface of SNF [29]. Prior to COF growth, SNF was aminated by immersing it in 5% (*v*/*v*) 3-aminopropyltriethoxysilane (APTES, Shanghai Titan Scientific Co., Ltd., Shanghai, China) ethanol solution at room temperature for 12 h. After the reaction, the membrane was washed thoroughly with absolute ethanol to remove residual APTES and dried at 70 °C for 12 h.

The aminated SNF membrane was then fixed in the center of a homemade H-type diffusion cell as the reaction substrate. The organic phase consisted of 1.1 mmol·L^−1^ 1,3,5-triformylphloroglucinol (TFP, Shanghai Titan Scientific Co., Ltd., Shanghai, China) dissolved in 1,3,5-trimethylbenzene (Shanghai Titan Scientific Co., Ltd., Shanghai, China), whereas the aqueous phase consisted of 1.7 mmol·L^−1^ 2,5-diaminobenzenesulfonic acid (DABA, Shanghai Haohong Biomedical Technology Co., Ltd., Shanghai, China) dissolved in deionized water. Acetic acid (Anhui Zesheng Technology Co., Ltd., Anqing, China) was added to the organic phase as the catalyst. The two phases were introduced into the two sides of the diffusion cell and reacted at 65 °C for 24 h. The resulting membrane was washed repeatedly with methanol (Sinopharm Chemical Reagent Co., Ltd., Shanghai, China) and deionized water and then dried under vacuum at 60 °C for 12 h. The product was designated as SNF@SCOF.

### 2.3. Preparation of SiO_2_@TpPa-SO_3_H/Fe (SNF@SCOF-Fe)

To prepare the Fe-doped composite membrane, the aminated SNF membrane was placed in the center of the H-type diffusion cell. The organic phase was prepared by dissolving TFP (1.1 mmol·L^−1^) in 1,3,5-trimethylbenzene, and the aqueous phase was prepared by dissolving DABA (1.7 mmol·L^−1^) and FeSO_4_·7H_2_O (1.0 mmol·L^−1^, Sinopharm Chemical Reagent Co., Ltd., Shanghai, China) in deionized water. Acetic acid was added to the organic phase as the catalyst. The two solutions were introduced into the two compartments of the diffusion cell and allowed to react at 65 °C for 24 h, during which interfacial diffusion enabled in situ growth of Fe-coordinated TpPa-SO_3_H on the aminated SNF surface. After the reaction, the membrane was collected, washed thoroughly with methanol and deionized water to remove residual monomers and loosely bound species, and dried before use. The final product was denoted as SNF@SCOF-Fe.

### 2.4. Characterization

X-ray diffraction (XRD) analysis was conducted using a PANalytical X’Pert3 powder diffractometer (Malvern Panalytical, Almelo, The Netherlands). The morphology and microstructure of the samples were examined using scanning electron microscopy (SEM, Zeiss Gemini 360, Carl Zeiss Microscopy, Oberkochen, Germany) and transmission electron microscopy (TEM, JEOL JEM-F200, Tokyo, Japan). UV–vis diffuse reflectance spectroscopy (DRS) was performed on a Shimadzu UV-2600 spectrophotometer (Shimadzu, Kyoto, Japan) equipped with an integrating sphere, using BaSO_4_ as a reflectance reference. Fourier-transform infrared spectroscopy (FTIR, INVENIO S, Bruker Corporation, Billerica, MA, USA) was used to identify functional-group changes after COF growth and Fe incorporation. X-ray photoelectron spectroscopy (XPS, ESCALAB 250Xi, Thermo Fisher Scientific, Waltham, MA, USA) was performed to evaluate the elemental composition, Fe oxidation state, and surface coordination environment of SNF@SCOF-Fe. Inductively coupled plasma optical emission spectroscopy (ICP-OES) was used to quantify Fe loading and Fe leaching. Hydroxyl radical generation was monitored using a fluorescence spectrophotometer (F-4600, Hitachi High-Tech, Tokyo, Japan; excitation: 310 nm, emission: 425 nm).

### 2.5. Photothermal Performance Evaluation

The photothermal performance of the samples was evaluated using an infrared thermal imaging camera (Teledyne FLIR E5xt, Teledyne FLIR, Wilsonville, OR, USA). SNF, SNF@SCOF, and SNF@SCOF-Fe were cut into circular pieces with a diameter of 1.0 cm and immersed in phosphate-buffered saline (PBS, pH 7.4). The samples were irradiated under an 808 nm NIR laser (Beijing BLUEPRINT laser, 1.0 W·cm^−2^, Beijing BLUEPRINT, Beijing, China), a 420 nm visible-light source (Beijing Perfectlight PLSSXE300+, equipped with a 420 nm bandwidth filter, 0.5 W·cm^−2^, Beijing Perfectlight, Beijing, China), or a dual-light source (420 + 808 nm). For the 808 nm laser, the irradiation spot was circular with a diameter of 1.5 cm, corresponding to an area of approximately 1.77 cm^2^. For the 420 nm light source, the irradiated square area had a side length of 2.0 cm, corresponding to an area of approximately 4.0 cm^2^. The sample-to-light-source distance was 12.5 cm. Thermal images and surface temperatures were recorded every 1 min during irradiation.

### 2.6. Detection of Reactive Oxygen Species

Reactive oxygen species (ROS) generation was assessed using specific chemical probes under 420 nm, 808 nm, and dual-light irradiation. Singlet oxygen (^1^O_2_) production was monitored by mixing a circular membrane sample (diameter: 1 cm) with 1 mL of 1 mM 1,3-diphenylisobenzofuran (DPBF, Adamas, Shanghai, China) in dimethyl sulfoxide (DMSO, Adamas, Shanghai, China) and recording the decay of its characteristic UV–vis absorption peak. Superoxide radical (·O_2_^−^) generation was evaluated analogously using 1 mL of 1 mM nitro blue tetrazolium (NBT, Adamas, Shanghai, China) solution, with changes in the NBT absorption profile serving as the detection metric. Hydroxyl radical (·OH) formation was quantified by incubating the membrane with 1 mL of 5 mM disodium terephthalate (NaTA, Adamas, Shanghai, China) solution and measuring the resultant fluorescence emission intensity (excitation: 310 nm; emission: 425 nm) at designated time intervals.

### 2.7. Antibacterial Assay

Supplier-reported MDR enteropathogenic *E. coli* (CICC 10663, Beijing, China) and *MRSA* (CICC 25138, Beijing, China) were selected as representative Gram-negative and Gram-positive model strains, respectively. Both strains were purchased from the China Center of Industrial Culture Collection (CICC). According to the supplier’s documentation, these two bacterial strains show defined antibiotic resistance profiles and were therefore selected as representative resistant strains for antibacterial evaluation in this study. The *E. coli* strain is resistant to gentamicin, ceftriaxone, sulfamethoxazole, trimethoprim, tetracycline, and amoxicillin, whereas the *MRSA* strain is resistant to methicillin and oxacillin. The bacteria were cultured in Luria–Bertani (LB, Bkmam, Changde, China) broth at 37 °C with shaking until the late logarithmic growth phase. The bacterial concentrations were adjusted to approximately 1 × 10^8^ CFU·mL^−1^ for *E. coli* and 1 × 10^7^ CFU·mL^−1^ for *MRSA*.

Before antibacterial testing, the membranes were cut into circular pieces (diameter: 1.0 cm) and sterilized under UV irradiation. Each membrane was incubated with 1.0 mL of bacterial suspension in a 48-well plate and exposed to different light conditions, including 420 nm, 808 nm, and dual-light irradiation (420 + 808 nm), for 15 min. A bacterial suspension without any sample served as the control group, and dark-treated sample groups were also included to exclude dark toxicity.

After treatment, the bacterial suspensions were collected, serially diluted tenfold, and 5 μL aliquots were dropped onto LB agar plates using the hanging-drop method. Each dilution was spotted onto agar plates and incubated at 37 °C for 12–18 h, photographs of the colonies on the plates were taken, and the number of colonies was counted using ImageJ software (V1.53t).

To evaluate the contribution of heating alone, bacterial suspensions were incubated for 15 min in a water bath programmed to reproduce the dual-light temperature–time profile of SNF@SCOF-Fe, with a comparable heating rate and a peak temperature of approximately 54–55 °C, in the absence of both light irradiation and photoactive material. Antibacterial activity was then assessed following the standard antibacterial assay procedures.

The bacterial concentration was calculated using:

N = *n* × 1000/V × D

where N is the bacterial concentration (CFU·mL^−1^), *n* is the number of colonies formed from a single droplet, V is the droplet volume (5 μL), and D is the dilution factor.

The antibacterial rate was calculated as:

Antibacterial rate (%) = [(Nc − Ne)/Nc] × 100%

where Nc and Ne represent the bacterial number of the control and experimental groups, respectively.

### 2.8. Cytocompatibility Evaluation

The cytocompatibility of the samples was evaluated using the CCK-8 assay with L929 mouse fibroblasts (Procell, Wuhan, China) and MC38 murine colon carcinoma cells (Procell, Wuhan, China). Cells were seeded in 96-well plates at a density of 8000 cells per well and cultured for 24 h. The culture medium was then replaced with the extracts of the corresponding samples, and the cells were further incubated for 24 h. Afterward, CCK-8 reagent (Beyotime, Shanghai, China) was added and the absorbance of each well was measured to determine relative cell viability. To better approximate the irradiation conditions used in the antibacterial assays, an additional dual-light cytocompatibility assay was performed by co-incubating cells with SNF, SNF@SCOF, or SNF@SCOF-Fe followed by 420 + 808 nm irradiation for 15 min. For this cell-safety experiment, the irradiation condition was adjusted so that the maximum temperature did not exceed 45 °C, which was selected as a conservative upper boundary for preliminary in vitro cell evaluation based on the temperature-dependent biological responses reported for photothermal-related biomedical applications [30].

### 2.9. Live/Dead Bacterial Staining

Live/dead bacterial staining was conducted to assess bacterial viability and membrane integrity after treatment. *E. coli* and *MRSA* suspensions were incubated with SNF@SCOF-Fe and treated under dark control, 420 nm, 808 nm, or dual-light irradiation conditions for 15 min. The treated bacteria were collected, washed with sterile 0.85% NaCl solution, and stained with the SYTO 9/PI Live/Dead Bacterial Double Stain Kit (Probelives, Suzhou, China) in the dark at room temperature for 15 min. The stained samples were then observed by fluorescence microscopy (Olympus IX51, Olympus Corporation, Tokyo, Japan). Live bacteria with intact membranes emitted green fluorescence, while membrane-damaged dead bacteria exhibited red fluorescence.

### 2.10. Fe Leaching and Membrane Stability Tests

For Fe loading quantification, 10.06 mg of SNF@SCOF-Fe was digested and analyzed by ICP-OES (iCAP PRO, Thermo Fisher Scientific, Waltham, MA, USA). For the Fe leaching test, SNF@SCOF-Fe membrane samples were immersed in PBS for 1, 3, 5, and 7 days, and Fe concentrations in the supernatants were measured by ICP-OES. Soaking stability was evaluated by measuring the mass retention of SNF@SCOF-Fe after immersion in PBS for 1, 3, 5, and 7 days. Photothermal cycling stability was evaluated by irradiating the membrane under dual-light conditions for 15 min, allowing it to cool naturally to room temperature, and repeating this heating–cooling process for six cycles.

### 2.11. Statistical Analysis

Statistical analysis was performed using SPSS (V25.0) software. Comparisons between two groups were conducted using Student’s *t*-test and *p* < 0.05 was considered statistically significant.

## 3. Results and Discussion

**Scheme 1:**
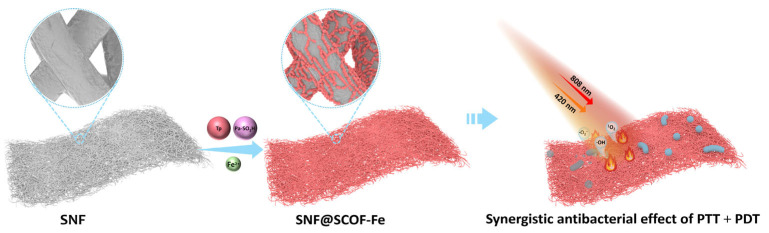
Schematic illustration of the fabrication process and the dual-light-triggered synergistic PTT/PDT antibacterial mechanism of SNF@SCOF-Fe.

As illustrated in Scheme 1, SNF@SCOF-Fe was fabricated through in situ interfacial growth of an Fe-coordinated sulfonated COF on electrospun silica nanofibers. Under dual-light irradiation (420 + 808 nm), the composite exhibits combined photothermal heating and ROS generation. The Fe-doped COF shell contributes to localized heating under irradiation while simultaneously producing multiple ROS (^1^O_2_, ·O_2_^−^ and ·OH). This combination is expected to enhance bacterial membrane damage and oxidative stress, thereby contributing to rapid inactivation of the resistant bacteria.

**Figure 1:**
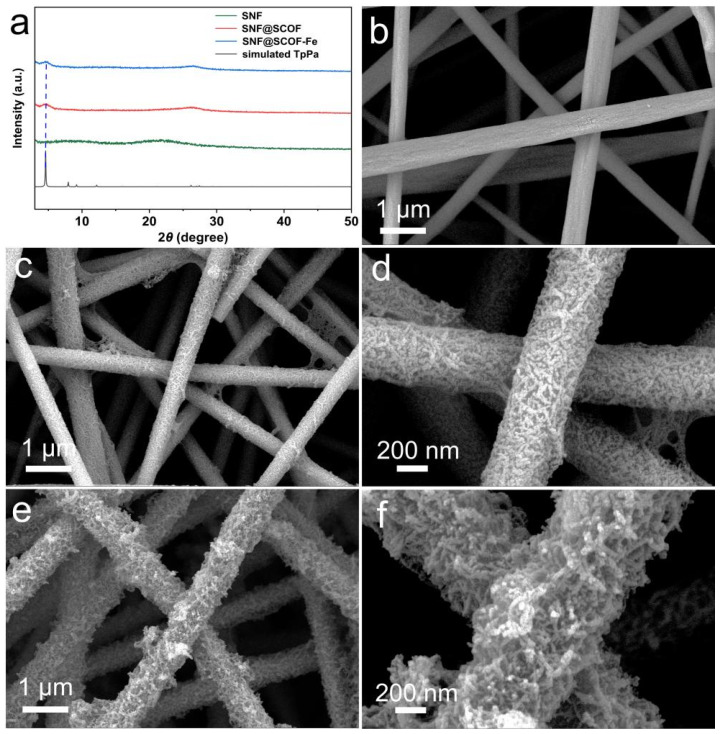
(**a**) XRD patterns of SNF, SNF@SCOF, and SNF@SCOF-Fe. (**b**) SEM image of SNF. (**c**,**d**) SEM images of SNF@SCOF. (**e**,**f**) SEM images of SNF@SCOF-Fe.

The XRD patterns of SNF, SNF@SCOF, and SNF@SCOF-Fe are shown in Figure 1a. Pristine SNF exhibits a broad diffraction peak centered at approximately 22.5°, which is characteristic of amorphous SiO_2_. After SCOF growth, a new diffraction peak appears at 4.7° for both SNF@SCOF and SNF@SCOF-Fe, indicating the successful formation of the TpPa-SO_3_H phase on the SNF surface [31]. No additional crystalline peaks are observed after Fe incorporation, and the overall peak positions and profiles remain nearly unchanged, suggesting that Fe species are highly dispersed within the composite rather than forming independent crystalline iron-based phases.

SEM images provide further evidence of the morphological evolution during membrane fabrication. As shown in Figure 1b, SNF consists of randomly oriented, smooth nanofibers forming a porous three-dimensional network, with diameters of approximately 300–600 nm. After SCOF deposition (Figure 1c,d), the fibrous framework remains intact, while the fiber surfaces become noticeably rougher and are covered by a dense nanoscale layer, indicating the successful growth of the COF coating. After Fe incorporation (Figure 1e,f), the overall fibrous morphology is preserved, whereas the surface roughness further increases, suggesting that the Fe-doping process does not compromise the structural integrity of the membrane.

**Figure 2:**
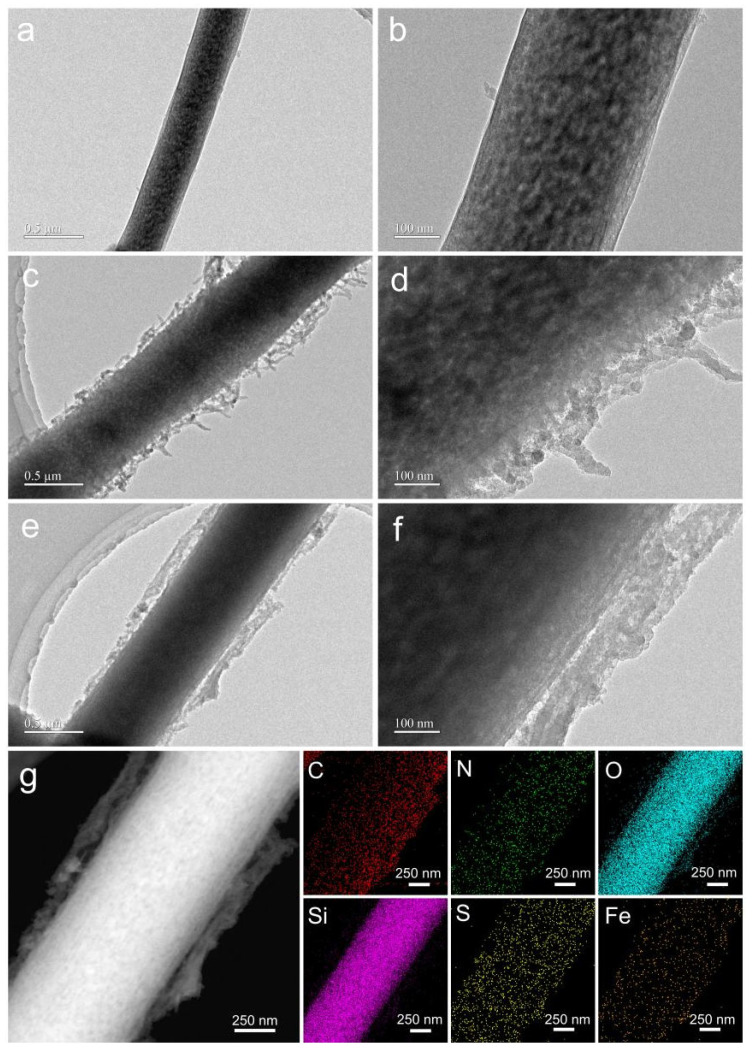
(**a**–**f**) TEM images of (**a**,**b**) SNF, (**c**,**d**) SNF@SCOF, and (**e**,**f**) SNF@SCOF-Fe. (**g**) HAADF-STEM image and corresponding elemental mapping of C, N, O, Si, S, and Fe for SNF@SCOF-Fe.

TEM analysis further reveals the hierarchical structure of the composite. Pristine SNF displays smooth and dense nanofibers (Figure 2a,b). After SCOF deposition (Figure 2c,d), a relatively uniform outer coating can be observed around the SiO_2_ fiber core, confirming the formation of a core–shell-like structure. After Fe incorporation (Figure 2e,f), the outer functional layer becomes more continuous and compact. In the HAADF-STEM image and elemental mapping (Figure 2g), Si is mainly distributed in the fiber core, whereas C, N, and S are uniformly distributed within the shell region, confirming successful deposition of the sulfonated TpPa COF. Importantly, Fe is also evenly distributed throughout the functional shell without obvious aggregation, indicating that Fe species are highly dispersed within the SCOF layer. Such homogeneous dispersion is expected to benefit both light harvesting and interfacial charge transfer.

To strengthen the evidence for Fe incorporation, XPS, FTIR, ICP-OES, and Fe-leaching analyses were performed. The XPS survey spectrum confirms the presence of C, N, O, Si, S, and Fe in SNF@SCOF-Fe (Figure S1). The high-resolution Fe 2p XPS spectrum further shows fitted Fe^2+^ peaks at 709.20 eV and 722.80 eV and Fe^3+^ peaks at 712.38 eV and 725.18 eV, confirming the coexistence of Fe^2+^ and Fe^3+^ species with predominant Fe^3+^ in SNF@SCOF-Fe (Figure S1f). FTIR spectra verify COF loading onto the SiO_2_ nanofibrous substrate and show characteristic C=C/C–N-related vibrations as well as spectral changes after Fe incorporation (Figure S2). ICP-OES analysis indicated an Fe loading of approximately 1.21 wt%. Moreover, Fe release from SNF@SCOF-Fe in PBS remained negligible over 7 days, with Fe concentrations of not detected (<0.001 ppm), 0.01, 0.03, and 0.13 ppm after 1, 3, 5, and 7 days, respectively (Table S1). These results confirm successful Fe incorporation and good Fe retention stability in the composite membrane.

**Figure 3:**
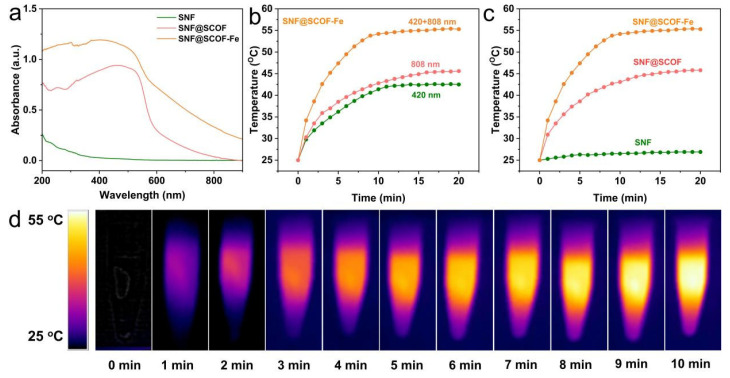
(**a**) UV-vis DRS spectra. (**b**) Photothermal heating curves of SNF@SCOF-Fe under different irradiation conditions. (**c**) Temperature profiles of various membranes under dual-light irradiation (420 + 808 nm). (**d**) Representative infrared thermal images of SNF@SCOF-Fe during dual-light irradiation.

The optical properties of the materials were investigated by UV-vis DRS. As shown in Figure 3a, SNF exhibits negligible absorption over the entire 200–900 nm range, reflecting the optical inertness of the silica substrate. In contrast, SNF@SCOF shows markedly enhanced absorption in the UV–visible region, especially between 400 and 500 nm, which can be attributed to the conjugated framework of TpPa-SO_3_H. After Fe incorporation, SNF@SCOF-Fe displays broadened and intensified absorption extending from the visible to the NIR region, indicating that Fe incorporation improves the light-harvesting capability of the system.

To evaluate the photothermal behavior, the temperature changes in the membranes under different irradiation conditions were monitored. As shown in Figure 3b, SNF@SCOF-Fe exhibits a larger temperature rise under dual-light irradiation than under either 420 or 808 nm irradiation alone. The membrane temperature increases rapidly from room temperature and reaches a plateau of 54.2 °C within approximately 10 min, indicating an evident dual-light-triggered heating response.

A comparison of different samples under dual-light irradiation further supports the role of Fe incorporation in the heating behavior (Figure 3c). SNF shows almost no temperature increase because of its poor light absorption, whereas SNF@SCOF reaches 45.8 °C after irradiation, indicating a moderate photoresponse. In contrast, SNF@SCOF-Fe exhibits the most pronounced temperature rise, reaching 55.1 °C after 20 min. Infrared thermal images (Figure 3d) reveal a rapid and spatially uniform heating process, which may help generate a controllable mild hyperthermic environment during antibacterial treatment. Although the temperature reached approximately 54–55 °C under dual-light irradiation, which is favorable for bacterial inactivation, this temperature range may also pose a potential risk of thermal injury to surrounding normal tissues. Thus, the present results should be regarded as an in vitro proof-of-concept demonstration, and further optimization of irradiation parameters is required to balance antibacterial efficacy and tissue safety. Moreover, given the limited tissue penetration of 420 nm light, the current dual-light configuration is more suitable for superficial or extracorporeal antibacterial applications than for deep-tissue infections.

**Figure 4:**
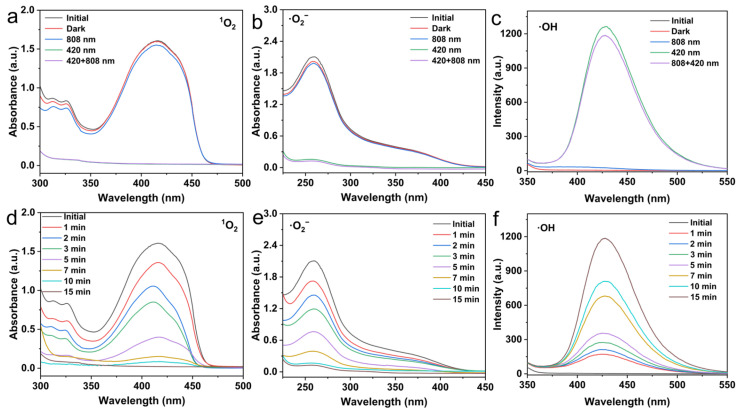
UV–vis absorption spectra of (**a**) DPBF and (**b**) NBT treated with SNF@SCOF-Fe under various irradiation conditions for 15 min. (**c**) Fluorescence spectra of NaTA for hydroxyl radical detection under various irradiation conditions for 15 min. Time-dependent spectra of (**d**) DPBF, (**e**) NBT, and (**f**) NaTA with SNF@SCOF-Fe under dual-light irradiation (420 + 808 nm) over 15 min.

The photodynamic capability of the composite membrane was further evaluated by monitoring the generation of multiple ROS under light irradiation. DPBF was used as a singlet oxygen probe. As shown in Figure 4a, SNF@SCOF-Fe induces substantial decay of the characteristic DPBF absorption under both 420 nm and dual-light irradiation, indicating effective ^1^O_2_ production. Under dual-light irradiation, the DPBF absorption continuously decreases with time and is nearly completely quenched after 15 min (Figure 4d), demonstrating sustained singlet oxygen generation.

Superoxide radicals were examined using NBT as the trapping agent. As shown in Figure 4b, SNF@SCOF-Fe causes significant attenuation of the NBT absorption under irradiation conditions involving 420 nm light, suggesting that visible-light excitation plays a dominant role in ·O_2_^−^ production. The time-dependent spectra under dual-light irradiation (Figure 4e) show that the absorption of NBT progressively decreases and is nearly exhausted after 15 min, confirming continuous superoxide radical generation.

Hydroxyl radical generation was evaluated using NaTA as a fluorescent probe. As shown in Figure 4c, Fe incorporation introduces a distinct ·OH-related fluorescence signal, indicating that Fe sites provide additional reactive centers for ·OH production. Under dual-light irradiation, the fluorescence intensity at 425 nm steadily increases with irradiation time (Figure 4f), suggesting cumulative generation of ·OH. Quantitative analysis of the time-dependent ROS probe responses was further performed to support the spectral observations shown in Figure 4d–f. Under dual-light irradiation for 15 min, the relative DPBF and NBT signals decreased rapidly to C/C_0_ values of 0.0132 and 0.0596, respectively, confirming efficient and sustained generation of ^1^O_2_ and ·O_2_^−^. In parallel, the normalized NaTA fluorescence intensity increased progressively to its maximum value, indicating continuous accumulation of ·OH during irradiation (Figure S3).

Additional control experiments were conducted to verify that the observed probe responses originated from light-triggered ROS generation by SNF@SCOF-Fe rather than probe instability, photobleaching, nonspecific adsorption, or dark reactivity. DPBF, NBT, and NaTA showed negligible signal changes in the probe-only dark, probe-only light, and membrane-probe dark control groups, confirming the reliability of the ROS detection assays (Figure S4). To further clarify the contribution of each component to ROS production, comparative experiments were performed using SNF and SNF@SCOF under dark, 808 nm, 420 nm, and dual-light irradiation conditions. SNF showed negligible generation of ^1^O_2_, ·O_2_^−^, and ·OH under all tested conditions, whereas SNF@SCOF produced evident ^1^O_2_ and ·O_2_^−^ under 420 nm-containing irradiation, with relatively stronger responses under 420 nm irradiation. However, ·OH generation from SNF@SCOF remained weak under both 420 nm and dual-light irradiation. These results indicate that the SCOF framework mainly contributes to visible-light-triggered ^1^O_2_ and ·O_2_^−^ generation, whereas Fe incorporation plays a critical role in promoting efficient ·OH production in SNF@SCOF-Fe (Figure S5).

**Figure 5:**
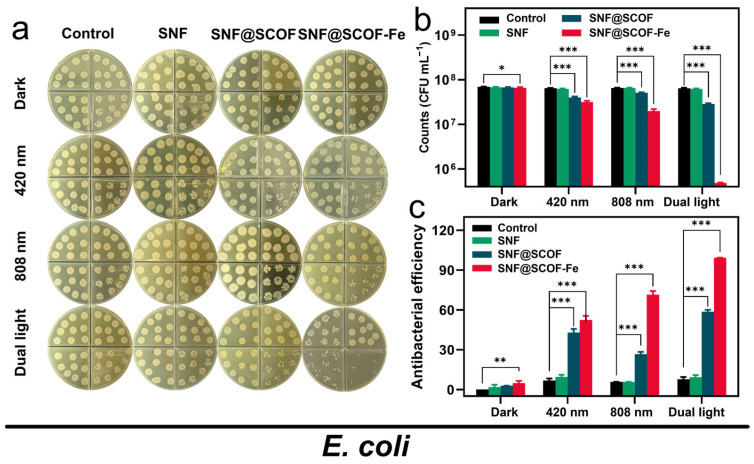
Antibacterial activity against *E. coli*. (**a**) Representative colony photographs, (**b**) corresponding bacterial concentrations, and (**c**) calculated antibacterial rates after 15 min of treatment under various irradiation conditions. Data are presented as mean ± SD (*n* = 3). * *p* < 0.05, ** *p* < 0.01, *** *p* < 0.001.

The antibacterial performance of the prepared membranes was first evaluated against drug-resistant *E. coli* under different irradiation conditions. As shown in Figure 5a, light irradiation alone did not induce a significant antibacterial effect in the absence of photoresponsive materials, confirming that the antibacterial activity mainly originated from the functional membranes rather than from the light source itself. After SCOF loading and Fe incorporation, both SNF@SCOF and SNF@SCOF-Fe exhibited enhanced antibacterial activity under light exposure, whereas SNF showed negligible bactericidal efficacy.

Under 420 nm irradiation for 15 min, the antibacterial rates of SNF@SCOF and SNF@SCOF-Fe against *E. coli* were 42.86% and 52.44%, respectively, indicating that the SCOF layer endowed the membrane with visible-light-triggered photodynamic antibacterial activity, while Fe incorporation further enhanced the overall effect. Under 808 nm irradiation for 15 min, the antibacterial rates of SNF@SCOF and SNF@SCOF-Fe were 26.66% and 71.43%, respectively. The higher antibacterial activity of SNF@SCOF-Fe under NIR irradiation is consistent with its stronger heating response after Fe incorporation. More importantly, under dual-light irradiation (420 + 808 nm) for 15 min, the SNF@SCOF-Fe group exhibited a pronounced reduction in bacterial colonies, with the bacterial concentration decreasing to the 10^5^ CFU·mL^−1^ level, markedly lower than that of the control group. The corresponding antibacterial rate reached 99.29%, far exceeding that of SNF@SCOF (58.65%) under the same conditions (Figure 5b,c).

These results indicate that SNF@SCOF-Fe can effectively inactivate *E. coli* through a dual-light-triggered cooperative mechanism. Specifically, visible-light excitation of the SCOF component promotes ROS generation, while NIR irradiation contributes to localized heating through the Fe-enhanced photothermal pathway. Mild hyperthermia may increase bacterial membrane permeability, thereby facilitating ROS penetration and amplifying oxidative damage. The combined action of these processes is consistent with the markedly improved bacterial inactivation observed for SNF@SCOF-Fe.

**Figure 6:**
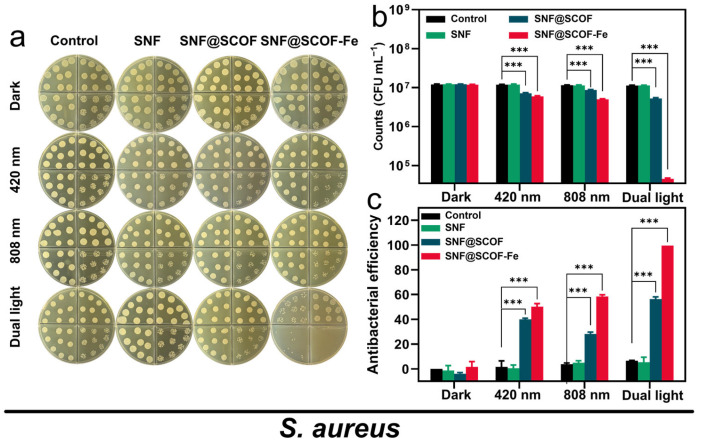
Antibacterial activity against *MRSA*. (**a**) Representative colony photographs, (**b**) corresponding bacterial concentrations, and (**c**) calculated antibacterial rates after 15 min of treatment under various irradiation conditions. Data are presented as mean ± SD (*n* = 3). *** *p* < 0.001.

The antibacterial performance of the composite membrane was further investigated using *MRSA* as a representative Gram-positive strain. As shown in Figure 6a, no obvious antibacterial effect was observed in the control group under light irradiation alone, again confirming that the antibacterial activity was material-dependent. Under 420 nm irradiation for 15 min, the antibacterial rates of SNF@SCOF and SNF@SCOF-Fe against *MRSA* were 39.99% and 50.25%, respectively. Under 808 nm irradiation for 15 min, the corresponding values were 28.17% and 58.56%, respectively. Similar to the results obtained for *E. coli*, SNF@SCOF-Fe displayed substantially stronger antibacterial activity than SNF@SCOF under NIR irradiation, further supporting the contribution of Fe incorporation to the heating response.

Under dual-light irradiation (420 + 808 nm) for 15 min, SNF@SCOF-Fe exhibited the most pronounced antibacterial effect, reducing the bacterial colony count by more than two orders of magnitude relative to the control group and achieving an antibacterial rate of 99.62% (Figure 6b,c). In contrast, SNF@SCOF showed a markedly lower antibacterial rate of 56.37% under the same conditions. The improved antibacterial performance of SNF@SCOF-Fe against both *E. coli* and *MRSA* indicates that the dual-light-triggered photothermal/photodynamic effect is effective against the two tested drug-resistant bacterial strains in vitro. It should be noted that the present antibacterial evaluation was limited to planktonic bacterial models. Given the relevance of biofilm-associated infections to antibacterial membrane applications, the lack of biofilm testing represents a limitation of this study. Future work should incorporate biofilm models to further evaluate the biomedical antibacterial potential of SNF@SCOF-Fe.

Combined with the ROS generation and photothermal results, the enhanced antibacterial performance of SNF@SCOF-Fe can be reasonably attributed to the following factors. First, the SCOF layer provides a visible-light-responsive photosensitizing platform capable of generating ROS. Second, Fe incorporation broadens the light absorption range into the NIR region and significantly improves photothermal performance. Third, Fe incorporation contributes to the formation of ·OH-related signals under light irradiation, as supported by the NaTA results, while the precise radical-generation pathway requires further mechanistic study. Finally, the electrospun silica nanofibrous substrate offers a flexible and porous support that facilitates uniform loading of the photoactive layer and intimate contact with bacterial cells. As a result, SNF@SCOF-Fe achieves efficient and rapid antibacterial action through integrated photothermal damage and oxidative stress under dual-light irradiation.

To quantitatively assess whether the dual-light effect exceeded simple additivity, the Bliss independence model was applied. For *E. coli*, the measured PDT and PTT inhibition values for SNF@SCOF-Fe were 52.44% and 71.43%, giving an expected additive inhibition of 86.41%, whereas the observed dual-light inhibition was 99.29%. For *MRSA*, the measured PDT and PTT inhibition values were 50.25% and 58.56%, giving an expected additive inhibition of 79.38%, whereas the observed dual-light inhibition was 99.62%. Because the observed values exceeded the expected additive values for both strains, the dual-light treatment showed a synergistic interaction under the tested irradiation conditions (Table S2).

A temperature-matched heating-only control was also performed to separate thermal killing from ROS-mediated antibacterial effects. In the absence of light irradiation and photoactive material, water-bath heating that reproduced the dual-light temperature–time profile of SNF@SCOF-Fe resulted in antibacterial efficiencies of 51.39% against *E. coli* and 43.36% against *MRSA*, both of which were lower than those achieved by SNF@SCOF-Fe under dual-light irradiation, at 99.29% and 99.62%, respectively (Figure S6). These results indicate that heating contributes to bacterial inactivation but does not fully account for the superior dual-light antibacterial performance. Qualitative live/dead bacterial staining further showed the strongest membrane damage in the dual-light-treated SNF@SCOF-Fe group for both *MRSA* and *E. coli*, consistent with the colony-counting results (Figure S7). A summary of membrane type, irradiation condition, temperature, ROS generation, antibacterial efficacy, and expected mechanism is provided in Table S3.

**Figure 7:**
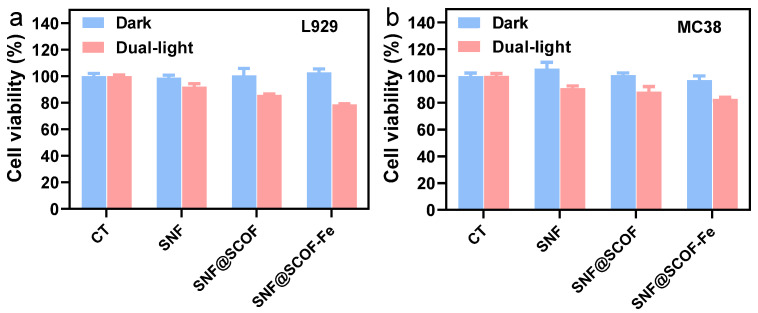
Cytocompatibility evaluation of the prepared membranes under dark conditions and controlled dual-light irradiation for 15 min. Relative viabilities of (**a**) L929 fibroblasts and (**b**) MC38 murine colon carcinoma cells after treatment with SNF, SNF@SCOF, or SNF@SCOF-Fe, as determined by the CCK-8 assay.

For antibacterial membrane applications, cytocompatibility toward relevant mammalian cells is a critical consideration in addition to antibacterial efficacy. Therefore, L929 fibroblasts and MC38 murine colon carcinoma cells were selected to evaluate the in vitro cytocompatibility of the membranes. The CCK-8 assay showed that SNF@SCOF-Fe did not induce obvious cytotoxicity under non-irradiated extract conditions. Since the antibacterial mechanism relies on light-triggered hyperthermia and ROS generation, an additional cytocompatibility assay was conducted under controlled dual-light irradiation. After dual-light irradiation, the viabilities of L929 cells treated with SNF, SNF@SCOF, and SNF@SCOF-Fe were 92.21%, 85.89%, and 78.76%, respectively, while the corresponding viabilities of MC38 cells were 90.92%, 88.25%, and 82.86% (Figure 7). These results suggest that SNF@SCOF-Fe maintains acceptable in vitro cytocompatibility under the tested irradiation conditions, in which the maximum temperature was kept below 45 °C. However, the reduced cell viability under dual-light exposure indicates the need for further optimization of irradiation parameters. Moreover, this in vitro condition should not be regarded as universally clinically tolerable, as thermal tolerance varies with tissue context and heat dissipation. Further ex vivo and in vivo studies are therefore required to define the safety window before biomedical translation.

The operational stability of SNF@SCOF-Fe was further examined under physiological-like soaking and repeated irradiation conditions. After immersion in PBS for 1, 3, 5, and 7 days, the membrane retained 98.67%, 98.65%, 98.22%, and 97.77% of its initial mass, respectively, indicating good structural stability in the aqueous environment (Figure S8a). In addition, repeated dual-light heating–cooling tests over six cycles showed nearly unchanged temperature profiles, demonstrating stable photothermal performance during cyclic irradiation (Figure S8b). These results suggest that SNF@SCOF-Fe possesses good soaking stability and photothermal stability under the tested in vitro conditions.

## 4. Conclusions

In summary, we successfully fabricated a flexible dual-light-responsive antibacterial membrane (SNF@SCOF-Fe) through in situ growth of an Fe-doped sulfonated TpPa COF on an electrospun silica nanofibrous membrane. Structural and chemical analyses confirmed a hierarchical core–shell morphology, with Fe species uniformly dispersed throughout the organic framework. Fe doping modification broadened optical absorption into the near-infrared region and enhanced the temperature rise in the membrane under irradiation. Under dual-light irradiation, the composite exhibited a rapid heating response together with sustained generation of multiple ROS. Bliss independence analysis indicated that the dual-light antibacterial effect exceeded the expected additive effect of PDT and PTT under the tested irradiation conditions. Temperature-matched heating controls and live/dead staining further supported the contribution of ROS-mediated oxidative damage beyond hyperthermia. SNF@SCOF-Fe achieved antibacterial rates of 99.29% against *E. coli* and 99.62% against *MRSA* within 15 min, while maintaining acceptable preliminary cytocompatibility under controlled irradiation conditions. These results indicate that rationally designed COF-based flexible membranes merit further study as in vitro light-responsive antibacterial materials, but biofilm testing, expanded bacterial panels, mechanistic validation, and ex vivo/in vivo safety and efficacy studies are still required before biomedical translation.

## Data Availability

The original contributions presented in this study are included in the article. Further inquiries can be directed to the corresponding authors.

## Supplementary Materials

The following supporting information can be downloaded at: https://www.mdpi.com/article/10.3390/pharmaceutics18060715/s1, Figure S1: XPS characterization of SNF@SCOF-Fe. (a) Survey spectrum; high-resolution spectra of (b) C 1s, (c) N 1s, (d) O 1s, (e) Si 2p, and (f) Fe 2p; Figure S2: FTIR spectra of SNF, SNF@SCOF, and SNF@SCOF-Fe; Figure S3: Time-dependent ROS generation by SNF@SCOF-Fe under dual-light irradiation (0–15 min). (a) DPBF degradation (C/C_0_), indicating ^1^O_2_ production; (b) NBT degradation (C/C_0_), indicating ·O_2_^−^ production; and (c) NaTA fluorescence enhancement (C/C_max_), indicating ·OH production; Figure S4: Stability and dark-reactivity controls for DPBF, NBT, and NaTA probes. Spectral changes of DPBF (a–c), NBT (d–f), and NaTA (g–i) under dark conditions, dual-light irradiation, and incubation with SNF@SCOF-Fe in the dark, respectively; Figure S5: ROS generation of SNF and SNF@SCOF under different irradiation conditions. UV-vis absorption spectra of DPBF and NBT treated with SNF (a,b) or SNF@SCOF (d,e), and fluorescence spectra of NaTA treated with SNF (c) or SNF@SCOF (f) after 15 min under dark, 808 nm, 420 nm, or dual-light irradiation; Figure S6: Antibacterial performance of the heating-only group against *MRSA* and *E. coli*. (a) Colony photographs of the control and heating groups (heating condition: water bath mimicking the temperature-time profile of SNF@SCOF-Fe under dual-light irradiation); (b) Quantitative antibacterial efficiency of the heating-only treatment; Figure S7: Fluorescence images of *MRSA* (a) and *E. coli* (b) treated for 15 min under control, 420 nm, 808 nm, and dual-light conditions. Live cells were stained green, and dead or membrane-damaged cells were stained red; Figure S8: Stability evaluation of the SNF@SCOF-Fe membrane. (a) Mass retention of the membrane after immersion in PBS for 1, 3, 5, and 7 days; (b) Photothermal cycling performance of the membrane over six consecutive dual-light irradiation on/off cycles; Table S1: Fe leaching analysis of SNF@SCOF-Fe in PBS measured by ICP-OES (The ICP-OES instrument detection limit was 0.001 ppm; “not detected” indicates values below this threshold. Each time point was measured once.); Table S2: Bliss independence analysis of the PDT/PTT antibacterial effects of SNF@SCOF-Fe; Table S3:.Summary of membrane type, irradiation condition, temperature, ROS detected, antibacterial efficacy, and expected antibacterial mechanism.
