# Supplementary material for: Dual-Light-Responsive Fe-Doped Covalent Organic Framework-Functionalized SiO2 Nanofibrous Membrane for Synergistic Photothermal and Photodynamic Inactivation of Multidrug-Resistant Bacteria
Source: Pharmaceutics. 2026 Jun 10;18(6):715. doi: 10.3390/pharmaceutics18060715 (PMC13305538; doi:10.3390/pharmaceutics18060715)
Supplement: Supplementary file 1 [file pharmaceutics-18-00715-s001.zip › pharmaceutics-4312262-supplementary.pdf]

Supporting Information

Dual-Light-Responsive Fe-Doped Covalent Organic Framework-Functionalized SiO_2 Nanofibrous Membrane for Synergistic Photothermal and Photodynamic Inactivation of Multidrug-Resistant Bacteria

Ting Zou [1], Lanlan Ni [1], Keqiang Xu [1,*] and Yi Chang [2,*]

[1] Key Laboratory for Advanced Technology in Environmental Protection of Jiangsu Province, Yancheng Institute of Technology, Yancheng, 224051, P.R. China; 234003011030@stu.ycit.edu.cn (T. Z.); 244003011038@stu.ycit.edu.cn (L. N.)

[2] Department of Clinical Laboratory, Affiliated Hospital 6 of Nantong University, Yancheng Third People's Hospital, Yancheng 224001, P.R. China;

* Correspondence: keqiangxu@ycit.edu.cn (K. X.), changyi@ntu.edu.cn (Y. C.)

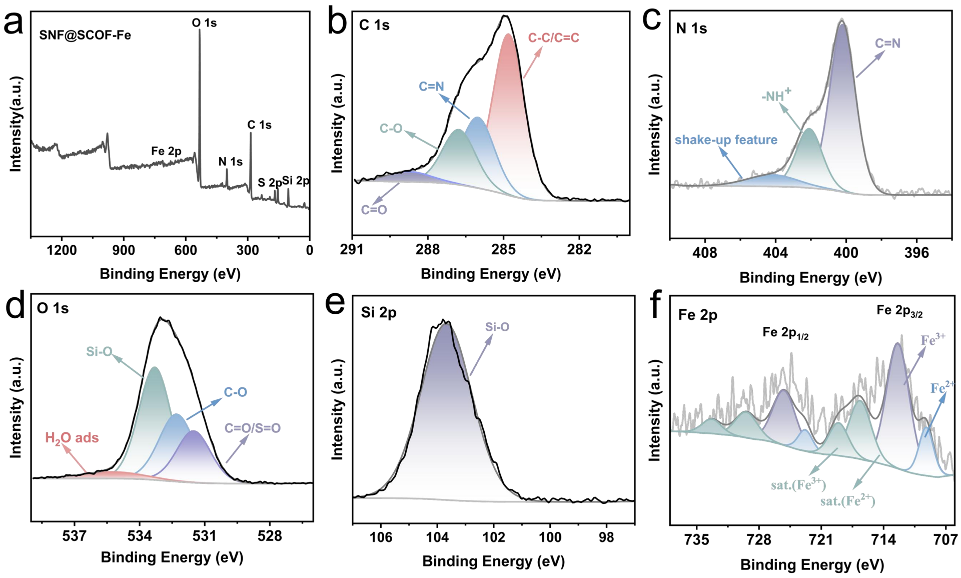

Figure S1. XPS characterization of SNF@SCOF-Fe. (a) Survey spectrum; high-resolution spectra of (b) C 1s, (c) N 1s, (d) O 1s, (e) Si 2p, and (f) Fe 2p.

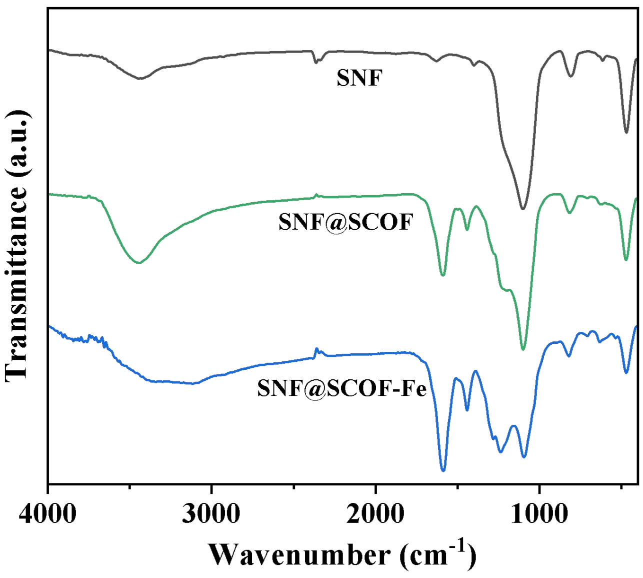

Figure S2. FTIR spectra of SNF, SNF@SCOF, and SNF@SCOF-Fe.

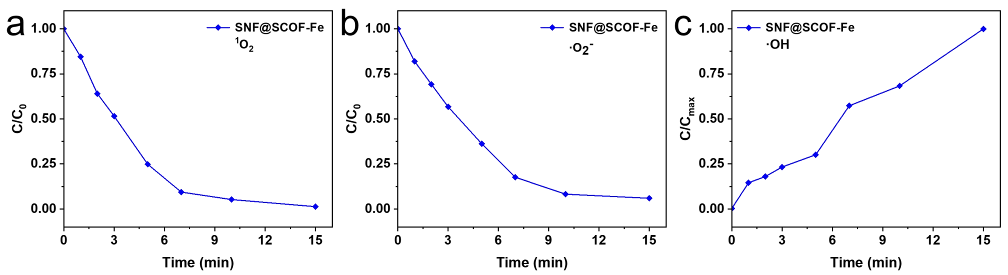

Figure S3. Time-dependent ROS generation by SNF@SCOF-Fe under dual-light irradiation (0-15 min). (a) DPBF degradation (C/C_0), indicating $^1O_2$ production; (b) NBT degradation (C/C_0), indicating $\cdot O_2^-$ production; and (c) NaTA fluorescence enhancement (C/C_{max}), indicating $\cdot OH$ production.

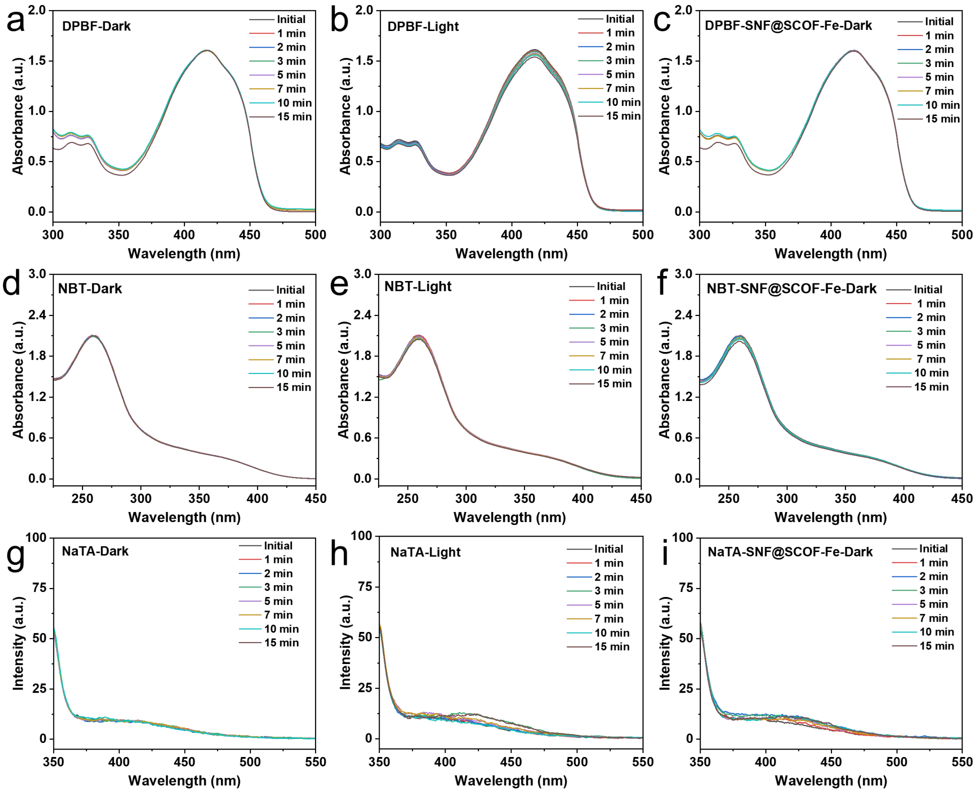

Figure S4. Stability and dark-reactivity controls for DPBF, NBT, and NaTA probes. Spectral changes of DPBF (a-c), NBT (d-f), and NaTA (g-i) under dark conditions, dual-light irradiation, and incubation with SNF@SCOF-Fe in the dark, respectively.

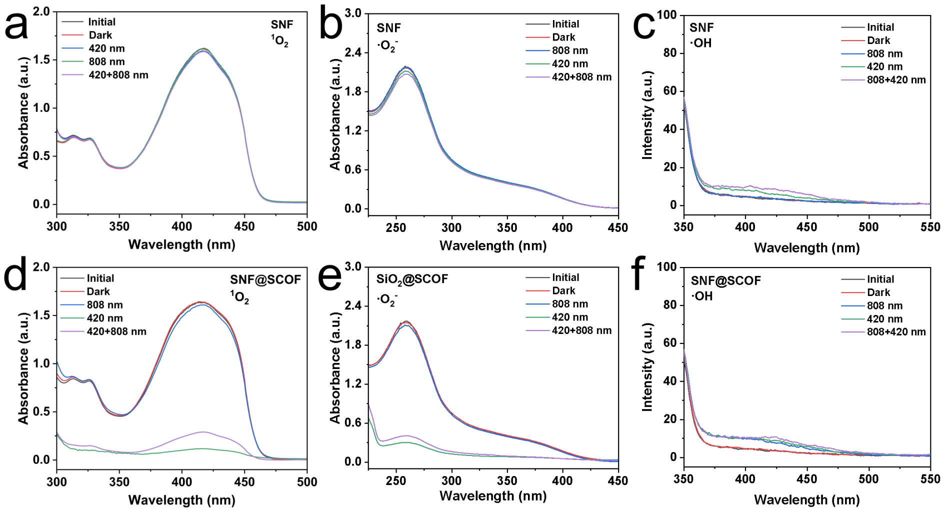

Figure S5. ROS generation of SNF and SNF@SCOF under different irradiation conditions. UV-vis absorption spectra of DPBF and NBT treated with SNF (a,b) or SNF@SCOF (d,e), and fluorescence spectra of NaTA treated with SNF (c) or SNF@SCOF (f) after 15 min under dark, 808 nm, 420 nm, or dual-light irradiation.

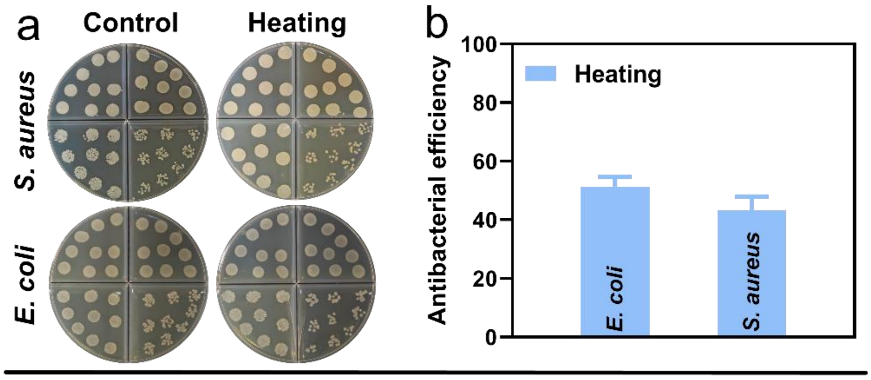

Figure S6. Antibacterial performance of the heating-only group against *MRSA* and *E. coli*. (a) Colony photographs of the control and heating groups (heating condition: water bath mimicking the temperature-time profile of SNF@SCOF-Fe under dual-light irradiation); (b) Quantitative antibacterial efficiency of the heating-only treatment.

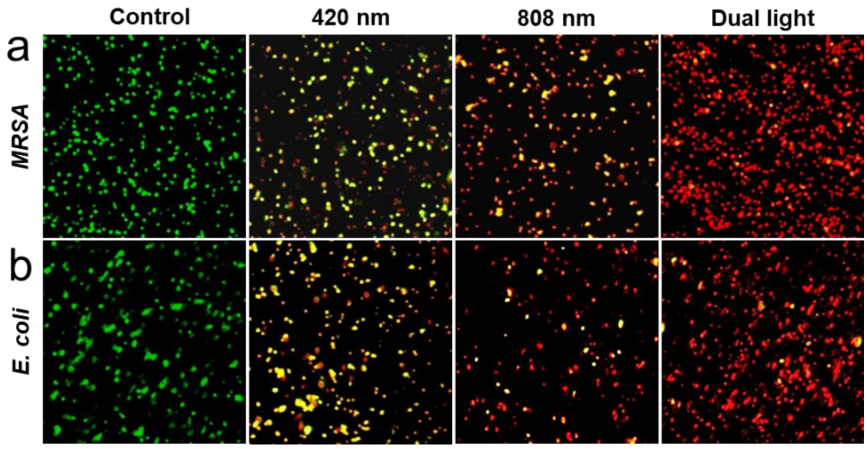

Figure S7. Fluorescence images of *MRSA* (a) and *E. coli* (b) treated for 15 min under control, 420 nm, 808 nm, and dual-light conditions. Live cells were stained green, and dead or membrane-damaged cells were stained red.

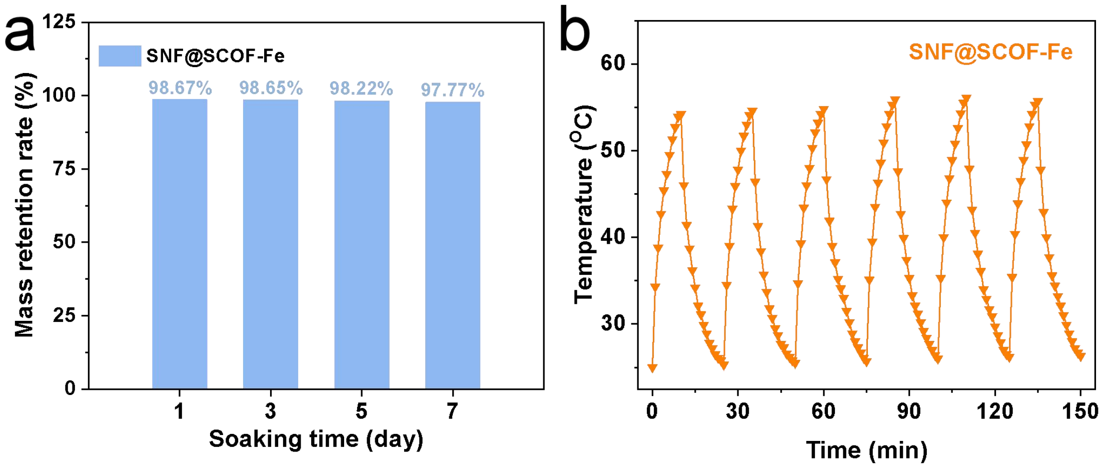

Figure S8. Stability evaluation of the SNF@SCOF-Fe membrane. (a) Mass retention of the membrane after immersion in PBS for 1, 3, 5, and 7 days; (b) Photothermal cycling performance of the membrane over six consecutive dual-light irradiation on/off cycles.

Table S1. Fe leaching analysis of SNF@SCOF-Fe in PBS measured by ICP-OES (The ICP-OES instrument detection limit was 0.001 ppm; "not detected" indicates values below this threshold. Each time point was measured once.)

Immersion time in PBS	Fe concentration in supernatant
1 day	Not detected
3 days	0.01 ppm
5 days	0.03 ppm
7 days	0.13 ppm

Table S2. Bliss independence analysis of the PDT/PTT antibacterial effects of SNF@SCOF-Fe

Bacterial Strain	Material	E_{PDT} (420 nm)	E_{PTT} (808 nm)	$E_{expected}$ (Calculated)	$E_{observed}$ (Actual)	Interaction Type
E. coli	SNF@SCOF-Fe	52.44%	71.43%	86.41%	99.29%	Synergistic
MRSA	SNF@SCOF-Fe	50.25%	58.56%	79.38%	99.62%	Synergistic

Table S3. Summary of membrane type, irradiation condition, temperature, ROS detected, antibacterial efficacy, and expected antibacterial mechanism.

Membrane type	Irradiation	Max temperature (15 min)	ROS species	Antibacterial efficacy (15 min)	Antibacterial mechanism
SNF	420 + 808 nm	26.8 °C	——	<10% (*E. coli*, *MRSA*)	——
SNF@SCOF	420 + 808 nm	45.2 °C	$^1O_2$;$\cdot O_2^-$	58.65% (*E. coli*), 56.37% (*MRSA*)	PDT
SNF@SCOF-Fe	DARK	25 °C	——	<10% (*E. coli*, *MRSA*)	——
	420 nm	42.4 °C	$^1O_2$;$\cdot O_2^-$; $\cdot OH$	52.44% (*E. coli*), 50.25% (*MRSA*)	PDT
	808 nm	44.9 °C	——	71.43% (*E. coli*), 58.56% (*MRSA*)	PTT
	420 + 808 nm	55 °C	$^1O_2$;$\cdot O_2^-$; $\cdot OH$	99.29% (*E. coli*), 99.62% (*MRSA*)	PDT + PTT
